# Data on LEGO sets release dates and worldwide retail prices combined with aftermarket transaction prices in Poland between June 2018 and June 2023

**DOI:** 10.1016/j.dib.2024.110056

**Published:** 2024-01-11

**Authors:** Wiktor Oczkoś, Bartosz Podgórski, Wiktoria Szczepańska, Tomasz Boiński

**Affiliations:** a Gdańsk University of technology, Faculty of Electronics, Telecommunication and Informatics, 11/12 Narutowicza Street, 80-180 Gdańsk, Poland; b Nicolaus Copernicus University in Toruń, Faculty of Economic Sciences and Management, 13a Gagarina Street, 87-100 Toruń, Poland

**Keywords:** Price history, Customer behavior, Price change prediction, Collector items

## Abstract

The dataset contains LEGO bricks sets item count and pricing history for AI-based set pricing prediction. The data spans the timeframe from June 2018 to June 2023. The data was obtained from three sources: Brickset.com (LEGO sets retail prices, release dates, and IDs), Lego.com official web page (ID number of each set that was released by Lego, its retail prices, the current status of the set) and promoklocki.pl web page (the retail prices for Poland, prices from aftermarket transactions). The data was merged based on the official LEGO set ID. With high granularity of the data (averaged monthly prices per LEGO set) the dataset permits the computation of variables at the set level and could support both aggregate and time-series analyses whereas the sparseness of the data permits the analysis of collector behavior allowing pinpointing of expected qualities from the purchased products and their resale potential. This may be useful to a broad range of researchers and data scientists using statistical methods and machine-learning techniques for price prediction.

Specifications Table

**Table:** 

Subject	Asset Pricing, Artificial Intelligence
Specific subject area	LEGO bricks sets item count and pricing history for AI-based set pricing prediction
Data format	Filtered, Aggregated
Type of data	Table (XLSX file)
Data collection	For downloading the base information from Brickset.com we used built-in API. The data was downloaded year by year with a pageSize parameter equal to 1000 as the API imposes limits on the number of requests per day. The data from lego.com and promoklocki.pl was implemented using a web scrapper that analyzed the webpages and extracted relevant information. The aftermarket transactions were limited to the range from June 2018 to June 2023. The data from all three sources were merged based on the Lego set ID and urlRetailPriceHistoryPLN.
Data source location	The retail prices present release prices of LEGO sets in the United States of America, United Kingdom, Canada, Germany, and Poland. The aftermarket prices come from the platform located in Poland. The data was obtained from three sources: Brickset.com (LEGO sets retail prices, release dates, and IDs), Lego.com official web page (ID number of each set that was released by Lego, its retail prices, the current status of the set) and promoklocki.pl web page (the retail prices for Poland, prices from aftermarket transactions).
Data accessibility	Repository name: BRIDGE of Knowledge (MOST Wiedzy) Data identification number: 10.34808/s25h-sx91 Direct URL to data: https://mostwiedzy.pl/pl/open-research-data/data-on-lego-sets-release-dates-and-retail-prices-combined-with-aftermarket-transaction-prices-betwe, **10210741381038465-0**

## 1 Value of the Data

• This data provides aggregated information on LEGO sets with their initial retail prices and their change through recent years showing owners' and collectors' behavior.
• With multiple sets released each year, there is uncertainty about the predicted price change of a given set in relation to the set contents, theme, and release year.
• This data will be of particular interest to investors trying to secure ownership of sets with higher revenue potential and researchers trying to analyze customer behaviour and predict price changes.
• The granularity of the data (averaged monthly prices per LEGO set) permits the computation of variables at the set level and could support both aggregate and time-series analyses. This may be useful to a broad range of researchers and data scientists using statistical methods and machine-learning techniques for price prediction.
• The sparseness of the data permits the analysis of collector behaviour allowing pinpointing of expected qualities from the purchased products and their resale potential.
• The created dataset covers a significant time frame which allows potential for longitudinal studies. This could be useful for analysis of the collectibles market evolvution over time.

## 2 Background

Statistical analysis of customer behaviour for collector's items, especially price prediction, is an actively researched problem by multiple groups and is an interesting area for future study and development [1], [2], [3]. Creation of either statistical or machine learning-oriented solutions requires, however, easy access to representative data sets from the field of interest. In the case of LEGO sets aftermarket LEGO bricks aftermarket investment grows each year showing annual growth from 15 to 30% [4]. The dataset is, however, not freely available and is mostly in the form of web pages requiring manual browsing or scrapping [4,5]. We thus believe that such a dataset, aggregating data about LEGO sets, like price, number of pieces, theme, etc. is useful for researchers wanting to create prediction mechanisms. The dataset might be also useful for people who are interested in investing in LEGO sets to help them choose the best option to invest. Although the aftermarket prices are given in Polish złoty, the prices correspond to those in global markets, as LEGO prices are standardized globally and LEGO collectors operate on global, not local markets. Furthermore the globality of the collectors market makes the price change trends applicable to any local market, as the prices of the given sets change similarly in every region.

## 3 Data Description

The data is composed of one aggregated table stored in an XLSX file named lego_final_data.xlsx. All data was scrapped from lego.com, brickset.com, and promoklocki.pl websites. The table contains the following columns:

• setID – internal Brickset.com LEGO set identification number,
• number – official LEGO set ID,
• numberVariant – official LEGO set sub variant (e.g. different minifigure hidden in a random bag),
• name – official LEGO set name,
• year – the set release year,
• theme – official name of the set theme,
• themeGroup – official name of the set themes grup (if available),
• subtheme – official name of the set sub-theme (if available),
• category – brickset.com internal set type,
• released – indicates whether the set was officially released (1) or not (0),
• pieces – number of parts in the set,
• minifigs – number of minifigures in the set,
• ownedBy – number of brickset.com users claiming that he or she owns the set,
• wantedBy – number of brickset.com users claiming that he or she wants to buy the set,
• rating – average set rating according to brickset.com users,
• reviewCount – number of the set reviews written by brickset.com users,
• packagingType – type of packaging for the set (if specified),
• availability – indicates whether the set was available in retail shops or only on official LEGO shop web site,
• instructionsCount – number of books with building instructions added to the set,
• minAge – LEGO recommended minimal user age for the set,
• maxAge – LEGO recommended maximal user age for the set (either not specified or 99),
• tags – list of brickset.com assigned set tags,
• LastUpdated – the date and time of the last update of the data in brickset.com in ISO 8601 format,
• urlRetailPriceCheckPLN – URL where retail price in PLN was downloaded from,
• US_retailPrice – retail price in United States in US dollars,
• US_dateFirstAvailable – date and time when the set became available in United States in ISO 8601 format,
• US_dateLastAvailable – date and time when the set stopped being officially available in United States in ISO 8601 format,
• UK_retailPrice – retail price in United Kingdom in GBP,
• UK_dateFirstAvailable – date and time when the set became available in United Kingdom in ISO 8601 format,
• UK_dateLastAvailable – date and time when the set became available in United Kingdom in ISO 8601 format,
• CA_retailPrice – retail price in Canada in Canadian dollars,
• CA_dateFirstAvailable – date and time when the set became available in Canada in ISO 8601 format,
• CA_dateLastAvailable – date and time when the set became available in Canada in ISO 8601 format,
• DE_retailPrice – retail price in Germany in EUR,
• DE_dateFirstAvailable – date and time when the set became available in Germany in ISO 8601 format,
• DE_dateLastAvailable – date and time when the set became available in Germany in ISO 8601 format,
• PL_retailPrice – retail price in Poland in PLN,
• Date – year and month for which the PriceMonthPLN is given,
• priceMonthPLN – price in PLN read from promoklocki.pl for year and month specified in Date column,
• status – official status of the set (if available) in LEGO web shop,
• urlRetailPriceHistoryPLN – URL containing retail and aftermarket price changes from the day of the release of the set, in PLN.

## 4 Experimental Design, Materials and Methods

The aim of our study was to create a comprehensive dataset about LEGO sets, including not only set attributes but also their price history on a monthly basis from June 2018 to June 2023. The primary motivation for conducting that research was the absence of a free dataset with such information. Usual such datasets contain only set details. The entire work was carried out on our own computers and using the Python language. For data processing, we chose the Pandas library. All the code described in this section can be found in the Lego_Predict GitHub public repository [6]. To reduce data noise, the intermediate XLSX files mentioned in this section were not added to the final dataset. They are, however, available in the GitHub repository alongside the code files. The column names used in the files are identical to the ones in the final dataset as described in the previous section.

The first step that we identified was obtaining data about the contents of the sets. During the review of available data, we managed to find four such sources. We compared them, and the results are presented in Table 1.

**Table 1:** The compare of data sources for Lego sets.

Name	Is it free?	Has it API?	Does it have good documentation?
Brickset	Yes	Yes	Yes
BrickEconomy	No	Yes	No
BrickLink	No	Yes	No
BrickOwl	Yes	Yes	No

As we can see, the best source turned out to be Brickset due to its rich API documentation and versatility of use. To obtain data, we only needed to create an account and obtain an API token. However, this source has one fundamental limitation. With one token, we can only make 100 requests per day. With such a limit, dependent on the type of query, to retrieve information about sets individually by ID over 20,000 requests would have to be done, by theme, there would be over 200 requests, or by years there would be about 80 requests. We decided to go by the last option. Another issue during this process was the default setting of the API ``pageSize'' parameter set to 500. It is responsible for the number of sets retrieved per page. Due to the error in the API, this default setting caused half of the sets to disappear from the result set. To solve this problem, we decided to set the parameter to 1000 (the value was obtained experimentally) which solved the problem of missing sets. The code used to obtain the information from this stage can be found in the ``brickset_download.py'' python file. Intermediate data from this step can be found in the ``lego_brickset.xlsx'' file.

The next step with the dataset obtained in this way was to retrieve data about availability statuses from the official lego.pl website. These statuses are one of the main factors that, in our opinion, affect the prices of the sets. In our work, we collected statuses in the Polish language; however, later in the study, they were translated into the English language based on official English status codes. The statuses can be found on the lego.com website [1], they are however available only as part of the HTML code of pages describing each set. They were extracted using the ``BeautifulSoup'' Python package due to its versatility in processing HTML code. To speed up the downloading process, we chose to check the statuses only for sets released after 2014. All older sets were marked as ``Retired Product'' as none of them are in sale anymore. During the download, we based our efforts on the official ID numbers assigned by Lego. Additionally, we had to implement protection against ``empty'' requests. Sometimes, a set had already had a subpage on Lego.pl, but there was no data there due to its potential discontinuation. It also happened that some sets never appeared on the Lego.pl website because they were released as part of various other initiatives, such as the ``Bricklink Designer Program'' [2]. To catch these errors, we decided to add a ``lack_in_system.txt'' file, where information about these sets can be found. The code needed to download statuses is located in the ``status_download.py'' python file, and the data with statuses can be found in the ``lego_status.xlsx'' file.

The third and final source of data we decided to utilize is the promoklocki.pl website. This website collects information about set prices in various online stores in Poland (prices in PLN). In our opinion, it is the most comprehensive compared to the competition. Furthermore, it stores historical data about price fluctuations, which was crucial in our study. Within this source, we had to perform three operations.

The first operation was to obtain links for historical data for each set and the recommended prices by Lego. For this purpose, we processed the HTML code using the ``BeautifulSoup'' Python package. The code is available in the ``links_download.py'' python file. The results of these operations are contained in ``lego_links.xlsx.''

With the obtained links, we proceeded to retrieve manufacturer-recommended prices. Here, too, we applied processing using ``BeautifulSoup.'' However, sometimes it happened that a set was too new and had not yet been released for sale. In such cases, it received the value ``Too fresh set.'' In the absence of a set on the website, we assigned the value ``Lack of data.'' This step was done using the code in the ``retail_price_poland_add.py'' Python file, and its results can be found in ``lego_retail_price.xlsx.''

The final step needed was to obtain historical data on set prices. To achieve this, we used the links obtained in the first step and retrieved the prices based on these links. We aggregated them on a monthly basis by taking the maximum price within that time period. In cases where a set was not available in a given month, we entered the value 0.00. The final result of this step was a smaller file called ``lego_data_price_history.xlsx,'' which contained the date, price, and a link that would later be used for merging files. All the code needed to perform this step can be found in the ``history_price_add.py'' python file.

With the data obtained in this way, we moved on to the processing and merging stage. The most important operation within this was the merging of tables from the ``lego_retail_price.xlsx'' and ``lego_data_price_history.xlsx'' files. The merging was done based on the link pointing to the Lego set description. Then we proceeded to clean the table, remove duplicated columns, and change names to be more self-explanatory. Finally, we translated the availability statuses to English, as mentioned earlier. All of these operations were coded in the ``data_processing.py'' python file.

The final result of all these operations was published within the proposed dataset [7] in a file named ``lego_final_data.xlsx'', which can be used by researchers for further work.

## Limitations

None.

## Ethics Statement

The authors have read and followed the ethical requirements for publication in Data in Brief and are confirming that the current work does not involve human subjects, animal experiments, or any data collected from social media platforms. The authors used only data obtained from public sources without involvement of any third parties. The downloaded data is not copyrighted and the data belonging to the LEGO Group (LEGO set details) are publicly available catalogue data.

The data obtained from the brickset.com web page was downloaded using the provided API designed specifically for such operations. The Terms of Service of the web page dopes not forbid such action and does not contain any conditions that would limit the automated data collection and redistribution. The data available on the web page is gathered from other public sites, like lego.com, and contains official catalogue information.

The data from the lego.com webpage was scrapped as the site does not provide any API. The data downloaded contains only catalogue information released by the LEGO group itself. The Terms of Service of the web page dopes not forbid such action and does not contain any conditions that would limit the automated data collection and redistribution.

The data from the promoklocki.pl was also scrapped as the page does not provide any API. The data downloaded contains historical changes of the LEGO set prices as downloaded from multiple public commercial web marketplaces. The web page does not contain any Terms of service that would limit the automated data collection and redistribution.

## CRediT authorship contribution statement

**Wiktor Oczkoś:** Conceptualization, Methodology, Software, Resources, Validation. **Bartosz Podgórski:** Conceptualization, Methodology, Software, Resources, Validation, Writing – review & editing. **Wiktoria Szczepańska:** Conceptualization, Methodology, Software, Resources, Validation, Writing – review & editing. **Tomasz Boiński:** Data curation, Writing – original draft, Supervision, Project administration.

## Data Availability

Data on LEGO sets release dates and retail prices combined with aftermarket transaction prices between June 2018 and June 2023 (Original data) (Bridge of Knowledge Open Research Data)
